# HELP — Heidelberg decision aid for lung cancer patients: a randomized controlled clinical trial

**DOI:** 10.1186/s13063-023-07365-2

**Published:** 2023-05-25

**Authors:** Nicole Deis, Laura Unsöld, Anja Siegle, Johannes Krisam, Michael Thomas, Matthias Villalobos

**Affiliations:** 1 Department of Thoracic Oncology, Thoraxklinik Heidelberg, Heidelberg University Hospital, Translational Lung Research Center Heidelberg (TLRC-H), Member of the German Center for Lung Research (DZL), Röntgenstraße 1, D-69126 Heidelberg, Germany; 2 Institute of Medical Biometry, Heidelberg University Hospital, Im Neuenheimer Feld 130.3, 69120 Heidelberg, Germany

**Keywords:** Lung cancer, Shared decision-making, Decision aid, Decision coaching

## Abstract

**Background:**

Shared decision-making (SDM), which increases the patient’s well-being, adherence, and success of treatment, is becoming increasingly important in medicine and especially in oncology. To empower patients to participate more actively in consultations with their physicians decision aids have been developed. In non-curative settings, such as the treatment of advanced lung cancer, decisions differ substantially from the curative setting, as uncertain gains in terms of survival outcomes and quality of life have to be weighed against the severe side effects of treatment regimens. There is still a lack of tools developed and implemented for such specific settings in cancer therapy that support shared decision-making. The aim of our study is to evaluate the effectiveness of the HELP decision aid.

**Methods:**

The HELP-study is designed as a randomized, controlled, open monocenter trial with two parallel groups. The intervention consists of the use of the HELP decision aid brochure, accompanied by a decision coaching session. The primary endpoint is clarity of personal attitude as operationalized by the Decisional Conflict Scale (DCS) after the decision coaching. Randomization will be performed as stratified block randomization according to the characteristic of preferred decision-making at baseline with a 1:1 allocation.

The participants in the control group get usual care, i.e., the doctor-patient conversation takes place without preliminary coaching and deliberation about their preferences and goals.

**Discussion:**

Developing decision aids (DA) for (lung) cancer patients with limited prognosis should empower patients to address these aspects and include information about “Best Supportive Care” as a treatment option. Using and implementing the decision aid HELP can not only give patients the possibility to include their personal wishes and values in the decision-making process, but also raise the awareness of shared decision-making itself among these patients and their physicians.

**Trial registration:**

German Clinical Trial Register DRKS00028023. Registered on 8 February 2022.

## Administrative information

**Table:** 

**Title {1}**	HELP (Heidelberg Decision Support for Lung Cancer Patients) is a monocenter, prospective, interventional, controlled, open and randomized study to compare the efficacy of a decision aid accompanied by a decision-coaching for lung cancer patients facing a treatment decision.
**Trial registration {2a and 2b}**	DRKS00028023 (German Clinical Trial Register) [registered on 8 February 2022]
**Protocol version {3}**	Version 1.0 of 22 December 2021
**Funding {4}**	Funding for this trial has been provided by the independent and non-profit Bristol Myers Squibb-Stiftung Immunonkologie (immune-oncology foundation), Munich.
**Author details 5a**	Nicole Deis, Dipl. Psych.^1^, nicole.deis@ med.uni-heidelberg.de Laura Unsöld, MSc^1^, laura.unsoeld@med.uni-heidelberg.de Anja Siegle, MA^1^, anja.siegle@med.uni-heidelberg.de Johannes Krisam, Dr. MSc^2^, krisam@imbi.uni-heidelberg.de Michael Thomas, Prof. MD^1^, michael.thomas@med.uni-heidelberg.de Matthias Villalobos, MD^1^, matthias.villalobos@med.uni-heidelberg.de (corresponding author) (^1^ Department of Thoracic Oncology, Thoraxklinik Heidelberg, Heidelberg University Hospital, Translational Lung Research Center Heidelberg (TLRC-H), Member of the German Center for Lung Research (DZL), Röntgenstraße 1, D-69126 Heidelberg, Germany; ^2^ Heidelberg University Hospital; Institute of Medical Biometry; Im Neuenheimer Feld 130.3, 69120 Heidelberg, Germany)
**Name and contact information for the trial sponsor {5b}**	Bristol Myers Squibb-Stiftung Immunonkologie c/o Bristol-Myers Squibb GmbH & Co. KGaA Contact name: Ms. Viola von Elsner Address: Arnulfstraße 29, 80636 München. Email: viola.elsner@stiftung-io.org
**Role of sponsor {5c}**	This funding source had no influence on the design of the study and will not have any role during its execution, analyses or interpretation of the data, as well as in decisions to submit results.

## Introduction

### Background and rationale {6a}

Shared decision-making (SDM) is becoming increasingly important in medicine and especially in oncology [1–5]. In SDM patients and caregivers discuss possible treatment options and patient preferences before making a decision together. When medical decisions have significant consequences for the patient’s future life, SDM increases the patient’s well-being, adherence to, and success of treatment [1, 6]. Particularly challenging in this context are the constantly evolving therapeutic algorithms associated with new treatment options that often only show marginal benefits and have to be balanced with end-of-life decision-making.

Decisions in the course of treatment are influenced by the doctor-patient relationship [7, 8]. This can tend to be paternalistic, i.e., the physician in his role as an expert either assumes responsibility and decision-making for the patient or the patient wishes him to do so. The model of shared decision-making, in which the physician and patient decide together on the basis of the available information and the patient’s preferences, has positive effects for patients and professionals [5]. However, it can only be applied if both professionals and patients are willing to take the patient’s preferences into account and make the decision together. Often, patients do not perceive the decision-making situations in the course of the disease as such and, in view of their situation, speak of an “absence of alternatives” to the form of therapy/treatment suggested by the professionals. Under certain circumstances, the diagnosis may give rise to a feeling of powerlessness and loss of control on the part of the patient. Shared decision-making therefore does not always take place and, as a result, the patient’s values and goals are not considered during the course of treatment. This can result in a poorer quality of life, over- or under-provision of care, reduced adherence to treatment, and increased stress for patients and hospital staff [9–11].

To empower patients to participate more actively in consultations with their physicians, decision aids have been developed [6, 12, 13]. Decision aids provide support for the individualized weighing of different decision options by presenting the benefits and risks of the measures under consideration in an evidence-based and generally understandable manner.

#### Existing knowledge

Patients wish more and more to participate in medical decision-making. This wish has increased steadily over the past 30 years. In 22 of the studies published from 2000 to 2012 on decision-making in cancer treatment, 85% of patients expressed a need to participate in medical decisions during their treatment process (vs. 62.5% in studies published up to 2000) [5].

Cancer patients often have unmet needs for information and communication about the disease, its course and prognosis, treatment alternatives, and side effects of therapy besides the above-mentioned strong desire to actively participate in treatment decisions [14, 15]. Patients who have a decision aid, on the other hand, feel better informed, are more aware of their own values, and participate more actively in the decision-making process [6, 12]. Compared with “usual care,” they also develop better coping strategies during the course of the disease, report fewer worries and fears, have more concrete assessments of the probabilities of therapeutic success and side effects, and show greater satisfaction with the therapeutic decisions [1]. Especially in oncology, decision aids are of particular relevance due to the severity of the disease and the importance of the decisions for the further course of life.

#### Need for a trial

Decisions taken in non-curative settings differ substantially from those taken in curative settings, as uncertain gains in terms of survival outcomes and quality of life have to be weighed against severe side effects of treatment regimens. There is still a lack of tools developed and implemented for specific settings in cancer therapy that support shared decision-making. By using the newly developed decision aid HELP accompanied by the decision coaching, lung cancer patients can become aware of their wishes, preferences, and needs with regard to treatment decisions. The aim of our study is to evaluate the effectiveness of the Help decision intervention and add evidence on decision aids in the context of incurable lung cancer.

### Objectives {7}

The aim of HELP is to promote patient autonomy and competence and facilitate shared decision-making in the context of treatment decisions for lung cancer patients. Patients who had decision coaching with the decision aid HELP are expected to show more clarity in their personal attitude concerning the treatment decision and feel better prepared for and more involved in the decision. The sum score of the dimension “clarity of personal attitude” in the Decisional Conflict Scale (DCS) was therefore chosen as a primary outcome.

Secondary outcomes are the effects of the HELP intervention on patients’ self-efficacy assessed via the Decision Self-Efficacy Scale, their perceived preparation for and involvement in treatment decision-making (Preparation for Decision-Making Scale (PDMS-D) and Patient Involvement in Care Scale (PICS)) and patients’ emotional state (EQ-5D-5L and Hospital Anxiety and Depression Scale (HADS-D)).

### Trial design {8}

The HELP-study is designed as a randomized, controlled, open monocenter, two-arm superiority trial with two parallel groups and a primary endpoint of “clarity of personal attitude” as operationalized by the Decisional Conflict Scale (DCS) which will be collected before the decision event. Following the assumptions that the HELP intervention strengthens patient autonomy and their clarity of personal values in relation to the treatment decision, the described design of the two-arm, parallel goups, superiority trials was chosen. Since it can be assumed that the effect of the HELP intervention will differ depending on the participant’s decision style, stratified randomization according to the decision type was chosen in order to take this aspect into account in the group comparisons of the outcome analyses. Therefore, randomization will be performed as stratified block randomization according to the characteristic of preferred decision-making at baseline with a 1:1 allocation.

## Methods: participants, interventions, and outcomes

### Study setting {9}

HELP will be conducted at the Department of Thoracic Oncology, University Hospital Heidelberg, which is one of the largest lung cancer centers in Germany. Here, about 600 patients are newly diagnosed with metastatic lung cancer every year.

### Eligibility criteria {10}

The patients involved in the study are facing a treatment decision due to a newly diagnosed lung cancer or due to a disease progression.

Inclusion criteria for patients:

- At least 18 years old;
- Able to give consent;
- Good German language skills;
- Diagnosis of incurable lung cancer (stage IV) and stage IIIb and IIIc; and
- Willingness to participate in the study.

Exclusion criteria for patients:

- Younger than 18;
- Insufficient knowledge of German;
- In a health condition that does not permit conversation due to a vital threat;
- Limitations in cognitive function; and
- Unable to give consent.

#### Decision coaches

The decision coaches are nursing professionals and psychologists who are trained in decision support and play a supporting, but neutral role in the decision. As the training for decision coaches and the conversation guide are standardized, variations in the decision coaching conversations should be kept to a minimum.

#### Physicians

To enable the integration of the decision aid in physician-patient consultations, a training course on the application of the decision aid in the context of everyday clinical practice at the Thoraxklinik Heidelberg was developed and conducted with the professionals involved in the decision-making process. In addition to important information and exercises on how to conduct conversations in the context of shared decision-making within the training, application materials (e.g., a manual) were provided for the professionals.

### Who will gather informed consent? {26a}

Research Assistants will introduce the trial to patients with the help of an information sheet. Patients have the opportunity to discuss the trial with the research assistant and to ask questions, before the research assistant will obtain written consent from patients willing to participate in the trial.

### Additional consent provisions for collection and use of participant data and biological specimens {26b}

Not applicable as no ancillary studies will be conducted.

## Interventions

### Explanation for the choice of comparators {6b}

Patients may feel better prepared and thus more able to participate actively in decisions concerning their treatment when they get a decision coaching and/or decision aid before the consultation takes place. Therefore, usual care, i.e. standard consultation with the physician without decision aid and/or decision coaching, was chosen as comparator. Patients without additional decisional support are expected to participate less in the consultation.

### Intervention description {11a}

Eligible patients will be randomized in equal proportions between the intervention group (i.e., decision coaching with decision aid) and the control group (i.e., usual care/standard consultation) according to the characteristic of preferred decision-making at baseline (expressions: physician should decide; patient would like to decide alone; patient and physician should decide together).

#### Intervention group

The intervention consists of the use of the decision aid, accompanied by a decision coaching session.

##### Decision aid

The decision aid HELP consists of a brochure for defining information and decision-making needs, including the patient’s personal values, as well as an overview of the most common forms of therapy. Both components are also available in digitalized form as a web app (htttps://awhelp.herokuappcom/#/).

The brochure entitled “What is important to me for my treatment?” includes questions on the patient’s information needs (e.g., information on the disease, treatment options, and side effects) and a section with questions on the patient’s decision-making behavior, i.e., how or with whom they want to make the treatment decision (alone, with the doctor, with relatives). The brochure ends with a clarification of the patient’s personal values (value clarification) and a section for open questions for the physician.

The second brochure entitled “Overview of common forms of therapy” includes information about the process, effect, duration and effort, side effects, and advantages vs. risks of common therapies, i.e., palliative therapy, chemotherapy, radiotherapy, immunotherapies, and targeted therapies.

##### Decision coaching

The coaching takes place in person or, if necessary, by telephone or online. The coaching process follows the sections of the HELP decision aid: patient’s information needs, the preferred method of decision-making, and the clarification of central personal values.

After the coaching session and before the consultation with the physician, participants fill in the questionnaires (Decisional Conflict Scale, Decision Self-Efficacy Scale, Preparation for Decision Making Scale, EQ 5D, Hospital Anxiety and Depression Scale) before their consultation with the physician. After the consultation, the post-survey questionnaires are handed out or mailed to participants (Patient Involvement in Care Scale, EQ-5D, Hospital Anxiety and Depression Scale).

#### Control group

The participants in the control group get usual care, i.e., the doctor-patient conversation takes place without preliminary coaching and deliberation about their preferences and goals.

Before the consultation with their physician, participants fill in the pre-survey questionnaires (Decisional Conflict Scale, Decision Self-Efficacy Scale, EQ 5D, Hospital Anxiety and Depression Scale). After the consultation, the post-survey questionnaires are handed out or mailed to them (Patient Involvement in Care Scale, EQ-5D, Hospital Anxiety and Depression Scale).

### Criteria for discontinuing or modifying allocated interventions {11b}

Patients can leave the study at any time for any reason, without any consequences. If a patient’s health status declines and they are no longer able to answer the survey questions, they will be treated as a dropout. The same applies for participants who withdraw their consent for any other reason. Patient data collected up to that moment will not be included in the analysis.

### Strategies to improve adherence to interventions {11c}

The adherence to fill out the questionnaires before and after the conversation with the physician takes place is ensured by friendly reminders via phone call or mail. Intervention adherence, i.e., ensuring that patients show up for the arranged coaching session, is ensured by reminding patients via phone call one day ahead of the appointment.

### Relevant concomitant care permitted or prohibited during the trial {11d}

As the intervention does not consist of any form of medical treatment, there are no restrictions concerning medical treatments.

### Provisions for post-trial care {30}

No provisions for ancillary and post-trial care will be provided, due to the absence of any physical intervention and the shortness of study.

### Outcomes {12}

The main objective is to investigate the effectiveness of the developed decision support and decision coaching for lung cancer patients. Effects will be measured by the sum score of the dimension “clarity of personal attitude” (items 4-6) of the Decisional Conflict Scale (DCS) [16, 17] after the decision coaching.

In addition, the effects of the HELP intervention on patients’ self-efficacy, their perceived preparation for and participation in treatment decision-making, and the patients’ emotional state will be assessed.

#### Primary outcome

The primary outcome is the sum score of the dimension “clarity of personal attitude” in the Decisional Conflict Scale (DCS) after the decision coaching has taken place. The questionnaire is validated in German and shows good reliability [16, 17].

A clinically relevant difference of 10 points in the primary outcome between the two treatment groups was assumed to be appropriate for case planning.

#### Secondary outcomes

Secondary outcomes are the effects of the HELP intervention on patients’ self-efficacy, their perceived preparation for and involvement in treatment decision-making, and their emotional state. All questionnaires listed in Table 1 are validated and show good reliability (see item 18b).

**Table 1:** Outcomes and instruments

**Outcome**	**Instrument**
Values clarity subscore	Decisional Conflict Scale (DCS) [16]
Self-efficacy	Decision Self-Efficacy Scale (DSES) [18]
Preparation for decision-making	Preparation for Decision Making Scale (PDMS-D) [19]
Patient involvement	Patient Involvement in Care Scale (PICS) [20]
Quality of life	EQ-5D-5L [21]
Anxiety and depression	Hospital Anxiety and Depression Scale (HADS-D) [22, 23]

### Participant timeline {13}

Participation in the study includes the completion of two questionnaires for each participant (before and after the medical consultation at intervals of 1–4 weeks). The questionnaires will be handed over personally (including a stamped return envelope) or sent by e-mail. The time needed to answer the questionnaires is about 10 min.

In addition to the questionnaire survey, the application of the decision aid incl. decision coaching will be carried out with participants in the intervention group. This can take place, depending on the patient’s wishes, either in the clinic or at another location, by telephone or by video conference. The time frame is about 30–45 min for the decision aid and the accompanying conversation. Appointments are made in person, by phone, or by mail.

**Fig. 1:**
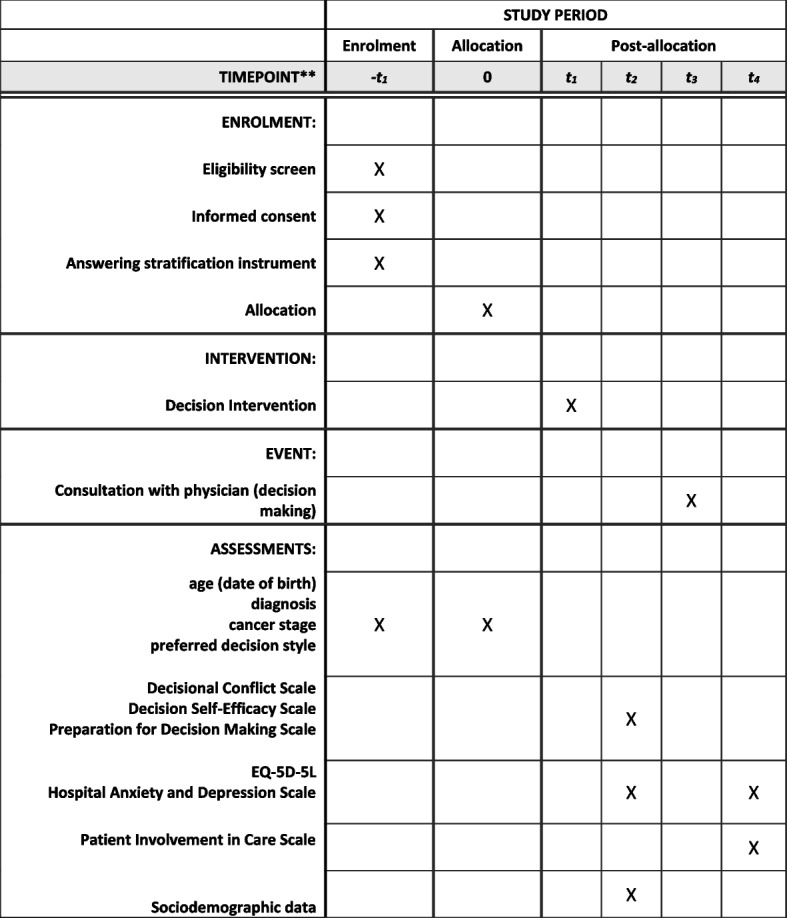
Schedule of enrolment, interventions, events, and assessments (SPIRIT figure). ** Timepoint: t_3_= consultation with physician (decision making), −t_1_ and t_0_ = 6–8 days before physician consultation, t_1_ and t_2_ = 2–5 days before physician consultation, t_4_ = 1–14 days after physician consultation

**Fig. 2:**
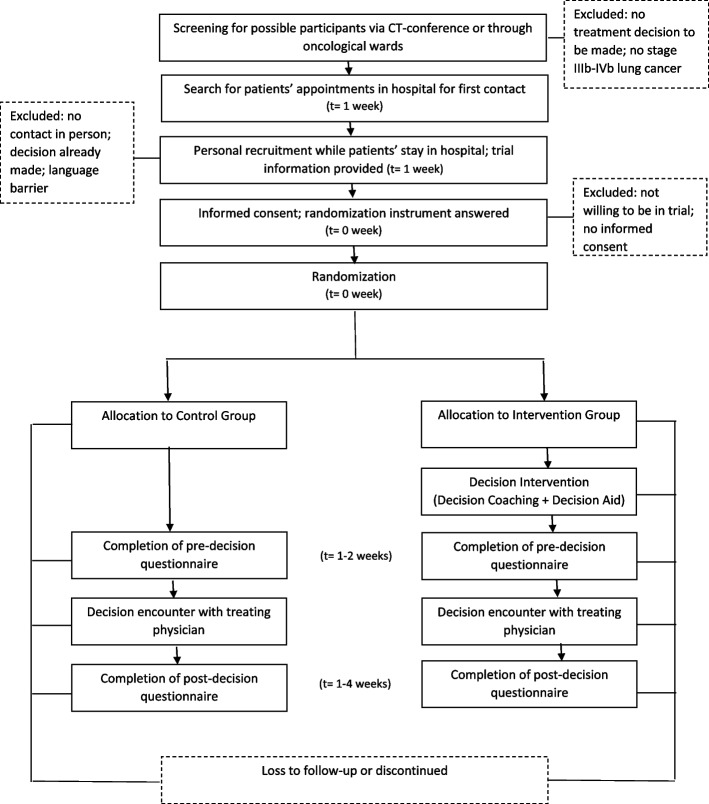
Participant timeline

The total study-related time required is therefore about 20 min for persons who participate exclusively in the questionnaire survey and about 50 to a maximum of 65 min for persons who are invited both to the questionnaire survey and to the decision coaching (Figs. 1 and 2).

### Sample size {14}

A clinically relevant difference of 10 points in the primary outcome between the two treatment groups was assumed to show a sufficient clinical difference for case planning. In addition, the standard deviation was estimated to be 20.6 from the literature [12]. Under these assumptions, *n*=71 patients per group are needed to demonstrate the assumed difference between the two groups at a two-sided significance level of 5% with a power of 80% using a Mann-Whitney *U* test. The calculation was performed by means of 100,000 simulation runs using the case number software PASS v 16.0.4. Taking into account a dropout rate of approx. 20%, 71/(1–0.20)=89 patients per group are included. These results in a total number of cases of *n*=178 patients are to be included in the study.

### Recruitment {15}

Patients are identified by the recruiting research assistants in CT- and indication conferences and tumor board meetings and on oncological wards. If patients meeting the eligibility criteria are identified, recruitment takes place when the patients are in the clinic for their examination appointments like MRT, bronchoscopy, etc. The expected recruitment rate is five to eight patients per week from March 2022 to December 2022.

The research assistants introduce themselves and the study to the patients and, if patients are willing to participate, they get the information sheet and sign the consent form. After that, randomization takes place.

## Assignment of interventions: allocation

### Sequence generation {16a}

Randomization to the two groups of the study (control and intervention group) will be performed in a ratio of 1:1. Stratified block randomization will be performed according to the characteristic of preferred decision-making at baseline (expressions: physician should decide; patient would like to decide alone; patient and physician should decide together). This is to ensure that participants are evenly distributed within the study groups according to their preferred decision style, since it is assumed that the intervention effect differs according to the individual differences in preferences for decision style.

### Concealment mechanism {16b}

Allocation concealment will be ensured by using opaque, sealed envelopes provided by the Institute of Medical Biometry, and takes place after the patients have been recruited.

### Implementation {16c}

After they have given written consent, participants are enrolled on the study. The recruiting research assistant opens the sealed envelopes and assigns patients to the intervention vs. control group according to the preferred decision-making style.

Participants in the control group receive the questionnaires and are instructed to return the filled-in questionnaires before their next consultation with their physician. Participants in the intervention group are informed that the decision coaching will take place before their next consultation and that they will be approached by the decision coach to make the appointment. The questionnaires are given to them after the decision coaching has taken place and before the consultation with the physician.

## Assignment of interventions: blinding

### Who will be blinded {17a}

Due to the design of the intervention, i.e., decision coaching with the decision aid, blinding is not possible, as the participants in the control group realize easily that they are not part of the intervention as they get no decision coaching and vice versa.

### Procedure for unblinding if needed {17b}

Since blinding is impossible (see above) there is no unblinding procedure.

## Data collection and management

### Plans for assessment and collection of outcomes {18a}

#### Primary outcome

The *Decisional Conflict Scale (DCS)* [16] was developed to operationalize the quality of medical decisions. It measures the uncertainty about which choice is the right one to make. The five dimensions are uncertainty, level of information, values clarity, social support, and effective decision.

For analysis, the authors recommend six sum scores: one for each of the five dimensions and one for an overall value. As the test construction assumes that the five subscales are predictors for the overall value, it is reasonable and in line with measurement theory to choose the subscale value clarity as the single primary outcome.

Both test-retest reliability (*r* ≥ .81) and internal consistency (Cronbach’s *α* ≥ .78) for all subscales are satisfactory. Cronbach’s *α* for the subscale values clarity is .91. Item selectivity and sensitivity for change are assumed to be satisfactory (effect sizes between 0.4 and 1.2) [16]. The DCS correlates with other constructs that are relevant for decision-making, e.g., decision regret, knowledge, or change of treatment decision. The German version of the DCS shows comparable values concerning internal consistency and correlations between subscales [17].

#### Secondary outcomes

The *Decision Self-Efficacy Scale (DSES)* [18] is based on Bandura’s concept of self-efficacy [24] and measures the perceived ability to engage in treatment-related behaviors and decisions. Cohen’s *α* is .84 and the scale discriminates adequately between patients who want to continue their treatment vs. patients that are unsure or prefer to delay the decision.

The *Preparation for Decision-Making Scale (PDMS)* is designed to assess the extent to which individuals feel prepared to make a medical decision with the help of supportive materials [25]. Preparation for decision-making is defined as “a patient’s perception of how useful a decision aid or other decision support intervention is in preparing the respondent to communicate with their practitioner at a consultation visit and making a health decision.” The Scale shows high internal consistency (*α* = .92) [25].

Results on the concurrent validity of the German version PDMS-D correspond with previous international study findings [26]. Individuals who felt well prepared reported lower decision uncertainty and less lack of information or social support, or ambiguity of personal values (subscales of the DCS) [19, 25].

The *Perceived Involvement in Care Scale (PICS)* is designed to examine the factors of doctor facilitation of patient involvement, level of information exchange, and patient participation in decision-making. All three subscales show good internal consistency (Cronbach’s α = .65–.87) [27].

In regard to construct validity, the subscales “patient activation by physicians” and “active information behavior of the patient” are highly correlated with satisfaction. The correlation of “active-information-behavior-of-the-patient” with satisfaction is positive but non-significant, which may be due to cultural differences in the healthcare systems in the US vs. Germany [20]. German patients tend to adhere to a paternalistic model and apparently expect stronger guidance from their physicians and tend to take less responsibility for medical decisions.

The *EQ-5D-5L* is an instrument for describing and valuing health. It defines health in 5 dimensions: mobility, self-care, usual activities, pain/discomfort, and anxiety/depression. The test-retest reliability (Cohens Kappa and/or ICC) and convergent validity for different patient populations have been shown to be good [28].

The *Hospital Anxiety and Depression Scale (HADS)* was designed to identify and quantify the two most common forms of psychological disturbances in medical patients, namely anxiety and depression. The Cronbach’s alpha and split-half reliabilities for both subscales of the HADS are .8 each. The HADS and its translations have been extensively validated [23, 29].

### Plans to promote participant retention and complete follow-up {18b}

Participants may withdraw from the study for any reason at any time. A drop-out rate of approximately 20% is expected. To keep drop-out at a minimal level, patients are reminded by a phone call to fill in the questionnaires and bring them to their next appointment.

### Data management {19}

The questionnaires will be checked for completeness and entered by qualified study personnel in the statistical analysis system. After data entry (according to the code book) a data quality check on plausibility will be performed. All questionnaires completed as part of the study will be digitized, archived at the Thoraxklinik after completion of the study, and retained for ten years. For the survey, patients are given a pseudonym. Only the study director has access to the pseudonym list of respondents. Only the digitized data will be shared for analysis. These electronic data are archived after completion and deleted after ten years.

### Confidentiality {27}

Only investigators will have access to study-related patient information. Personal information and confidentiality of data entry, coding, security, and storage are in line with the German privacy protection law (Bundesdatenschutzgesetz, BDSG) [30] and the privacy policy of the University Hospital Heidelberg.

All questionnaires completed as part of the study will be digitized, archived at Thoraxklinik after completion of the study, and retained for 10 years. For the survey, patients are given a pseudonym. Only the study director has access to the pseudonym list of respondents. Only the digitized data will be shared for analysis. These electronic data are archived after completion of the evaluation and deleted after 10 years.

### Plans for collection, laboratory evaluation, and storage of biological specimens for genetic or molecular analysis in this trial/future use {33}

As there will be no biological specimens collected there are no plans for laboratory evaluation and/or storage.

## Statistical methods

### Statistical methods for primary and secondary outcomes {20a}

A descriptive analysis of all collected patient information and data (as well as the scales that can be formed from the instruments) is performed: mean, standard deviation, median, minimum, and maximum, for continuous variables and absolute and relative frequencies for categorical variables.

For all (primary and secondary) analyses, the intervention arm (HELP intervention: decision coaching and decision aid) will be compared against the control arm (standard care/decision situation). The primary outcome measure is the sum score of the clarity of personal attitude dimension of the Decisional Conflict Scale (DCS). In addition to the descriptive evaluation, it will be analyzed by group comparisons (non-parametric comparison: van Elteren test) between the two study groups in which the stratification of randomization is considered.

For the sum scores and scales of the secondary outcomes, group comparisons (taking stratification into account) are also performed in order to detect significant differences. Depending on the group, the Cochran-Mantel-Haenszel test, the van Elteren test, or an analysis of covariance are used.

Results are reported along with descriptive *p*-values and 95% confidence intervals. *P*-values will be reported to four decimal places with *p*-values less than 0,05 considered statistically significant. Analysis is performed using statistical software SPSS (version 27, IBM) or R (version 4.0. http://r-pro-ject.org) (Table 2).

**Table 2:** Variables, measures, and methods of analysis

Variable/outcome	Hypothesis	Outcome measure	Methods of analysis
**1) Primary**	Intervention improved outcome in comparison to the control group		
Clarity of personal attitude/values clarity		Values clarity subscore of Decisional Conflict Scale (DCS-D)	van-Elteren-test
**2) Secondary**			
Decisional certainty	Improvement in comparison to control	Uncertainty subscore of Decisional Conflict Scale (DCS-D)	Cochran-Mantel-Haenszel test/ van-Elteren-test
Decisional support	Improvement in comparison to control	Support subscore of Decisional Conflict Scale (DCS-D)	Cochran-Mantel-Haenszel test/ van-Elteren-test
Being informed	Improvement in comparison to control	Informed subscore of Decisional Conflict Scale (DCS-D)	Cochran-Mantel-Haenszel test/ van-Elteren-test
Decision Effectiveness	Improvement in comparison to control	Effective decision subscore of Decisional Conflict Scale (DCS-D)	Cochran-Mantel-Haenszel test/ van-Elteren-test
Decreased decisional conflict	Improvement in comparison to control	Decisional Conflict Scale (DCS-D) – total score	Cochran-Mantel-Haenszel test/ van-Elteren-test
Self-efficacy	Improvement in comparison to control	Decisional Self-Efficacy Scale (DSES)	Cochran-Mantel-Haenszel test/ van-Elteren-test
Patient involvement in decision	Improvement in comparison to control	PDM subscore of Perceived Involvement in Care Scale (PICS-D)	Cochran-Mantel-Haenszel test/ van-Elteren-test
Information seeking	Improvement in comparison to control	PI subscore of Perceived Involvement in Care Scale (PICS-D)	Cochran-Mantel-Haenszel test/ van-Elteren-test
Health care provider facilitation	Improvement in comparison to control	HCP-FAC subscore of Perceived Involvement in Care Scale (PICS-D)	Cochran-Mantel-Haenszel test/ van-Elteren-test
Quality of life	Improvement in comparison to control	EQ5D-5L	Cochran-Mantel-Haenszel test/ van-Elteren-test
Emotional well-being	Improvement in comparison to control	Hospital Anxiety and Depression Scale (HADS-D)	Cochran-Mantel-Haenszel test/ van-Elteren-test
**3) Subgroup analyses**			
*Additional analysis IG*			
Preparation for decision	(Explorative approach)	Preparation for Decision Making Scale (PDMS-D)	Descriptive
**4) Sensitivity analyses**			
Per protocol analysis	Improvement in comparison to control	All outcomes	

### Interim analyses {21b}

No interim analyses will be performed.

### Methods for additional analyses (e.g., subgroup analyses) {20b}

The adjustment for the variable of decision-making type (used in the stratified randomization) is already considered with the application of the van-Elteren-test in the primary analysis of the outcome. There are no subgroup analyses planned.

### Methods in analysis to handle protocol non-adherence and any statistical methods to handle missing data {20c}

#### Analysis population

As analysis populations, the two common populations of intention-to-treat and per-protocol are defined and used. The intention-to-treat population considers all patients as randomized regardless of whether they finished the whole survey procedure. Criteria for determining the per-protocol population are as follows: patients who stayed in their randomized group, answered the first questionnaire before the decision-making (and for IG: after the intervention), and answered the second questionnaire within the timeframe of 1–4 weeks after the treatment decision.

#### Missing data

Missing data might occur by patients’ withdrawal from trial, unreturned questionnaires after the consultation, or other circumstances. Reasons for withdrawal will be reported for each randomization group and compared qualitatively. The effect that any missing data might have on results will be assessed via sensitivity analysis of augmented data sets. Dropouts and item nonresponse will be included in the analysis by imputation methods for missing data. Missing values in the primary outcome (and in other outcomes) are replaced by multiple imputation. Through an iterative series of predictive models using the non-missing variables in the dataset, the imputation is conducted. The iterations are run until it appears that convergence has been met. The accuracy of the imputations will depend on the information density in the dataset. Diagnostic plots are used to determine how valid the imputations may be. Imputation is conducted via predictive mean matching (PMM). PMM involves selecting a datapoint from the original, non-missing data which has a predicted value close to the predicted value of the missing sample. The closest values are chosen as candidates, from which a value is chosen at random. After the imputations are completed, all of the data (complete and imputed) will be combined and the analysis performed for each imputed and completed dataset.

### Plans to give access to the full protocol, participant level-data and statistical code {31c}

We are not able to provide data publicly due to the German data protection law and ethics approval regulations. Upon request, deidentified data can be shared, as can be the full protocol and statistical code.

## Oversight and monitoring

### Composition of the coordinating center and trial steering committee {5d}

This is a monocenter study designed, performed, and coordinated in the oncology department of the Thoraxklinik Heidelberg. The following responsibilities can be defined in the research team:

- Principal investigator: overseeing study design and preparation of protocol incl. revisions
- Lead investigator: responsible for the identification, recruitment, data collection and completion, data entry, and analysis
- Data manager: data verification and analysis
- Study coordinator: trial registration, annual reports

The study team meets weekly. No trial steering committee or stakeholder/public involvement group (nor any other committee) is involved.

### Composition of the data monitoring committee, its role and reporting structure {21a}

In accordance with EMA requirements, a Data Monitoring Committee is not needed as the study does not involve any medication, other medical treatment, or examinations such as blood samples, MRI, or X-ray examinations [31].

### Adverse event reporting and harms {22}

Participation in the study does not entail any health risks apart from the risk of infection with COVID-19 in the case of a face-to-face conversation. Study participants can choose between face-to-face or contact via telephone or video (e.g., Jitsi) to avoid the risk of infection. For face-to-face interview appointments, hygiene protection measures will be taken to mitigate the risk of infection. Filling out the decision aid and the accompanying preoccupation with personal information needs about the disease, personal values, and preferences, may put more psychological strain on patients than would otherwise be the case. It may also happen that patients in the control group, i.e., those who only fill in the questionnaires before and after the medical consultations, may perceive a psychological burden due to not receiving decision coaching.

As the study only involves questionnaires, there are no further risks. Blood samples, MRI, or X-ray examinations are not planned.

### Frequency and plans for auditing trial conduct {23}

No auditing of trial conduct is intended due to the shortness of the study and the absence of any kind of medical intervention.

### Plans for communicating important protocol amendments to relevant parties (e.g., trial participants, ethical committees) {25}

Any modifications to the protocol which may impact on the conduct of the study will require a formal amendment to the protocol. Modifications will have to be agreed upon by all members of the research team and approved by the Ethics Committee.

### Dissemination plans {31a}

The results will be made available in peer-reviewed journals and presented at conferences. Both positive and negative results will be reported. In addition, a “corona-compliant” hybrid symposium will take place, at which the results of the study will be presented to experts and the general public and an exchange of information about the current possibilities and limitations of shared decision-making in the context of (lung) cancer therapy will be facilitated.

## Discussion

Deficits in SDM are still evident in oncology practice and are most challenging when treatment options are limited and new treatment options only show marginal benefits. These deficits may lead to patients choosing a paternalistic decision-making model. Thus, individual wishes, values, and preferences may be neglected. We provide and evaluate a solution how to support SDM in the context of lung cancer. The expected results will contribute to the scientific discussion and expand knowledge of how to further investigate and develop SDM.

### Limitations

Another intervention group — “decision aid only”, i.e., patients working with the decision aid by themselves, without coaching — would be desirable. With regard to the low possibility of recruiting an additional 89 patients in the given time, the idea of a second intervention group was dismissed.

In the treatment of advanced lung cancer, there is no choice between several equivalent, evidence-based treatment options. Rather, patients have access to one single more or less promising treatment option vs. early palliative care alone or best supportive care. Our decision aid can therefore not be classified as a “traditional” decision aid according to the standards of evidence-based medicine. Patients have to decide between tumor-centered or symptom-oriented therapy and perceive a lack of alternatives concerning their treatment. Palliative care is often seen as giving in to their disease and consequently not an option to be considered.

Another limitation arises from the organizational context: When treatment decisions are, e.g., made by physicians in tumor boards, what is the relevance of patients’ preferences? It is of course still possible to make shared decisions, but are physicians and patients really open to that?

### Strengths

Our DA for (lung) cancer patients with limited prognosis empowers patients to address “Best Supportive Care” as a treatment option during consultations with their physicians. Using and implementing the decision aid HELP can not only give patients the possibility to include their personal wishes and values in the decision-making process, but also raise the awareness of shared decision-making itself among these patients and their physicians.

## Trial status

Protocol version 1.0, 22.12.2021; First enrolment: 10.03.2022; recruitment will be completed bv approx. 31.01.2023.

## Data Availability

All investigators involved in the study will have full access to the data. Any data required to support the protocol can be supplied on request, as there are no contractual agreements limiting access for investigators.

## Abbreviations

BDSG: Bundesdatenschutzgesetz
DA: Decision aid
DCS: Decisional Conflict Scale
DSES: Decision Self-Efficacy Scale
EMA: European Medicines Agency
EQ-5D-5L: European Quality of Life 5 Dimensions 5 Levels
HADS-D: Hospital Anxiety and Depression Scale, German Version
PDMS-D: Preparation for Decision Making Scale, German Version
PICS: Perceived Involvement in Care Scale; SDM: Shared Decision Making
